# Effects of Pulsed Electric Field on the Cell Wall and Infection Activity of *Rhizoctonia solani*

**DOI:** 10.3390/biology8040073

**Published:** 2019-09-26

**Authors:** Xuebin Feng, Peijun He, Chaoya Pan, Jin Xu, Baoshan Xue, Wenqing Yin, Yan Qian

**Affiliations:** College of engineering, Nanjing Agricultural University, Nanjing 210031, China; fxb9510@njau.edu.cn (X.F.); 30316416@njau.edu.cn (P.H.); pcynjau@sina.com (C.P.); xujin@njau.edu.cn (J.X.); 32216230@njau.edu.cn (B.X.); yinwq@njau.edu.cn (W.Y.)

**Keywords:** pulsed electric field, infection activity, chitin, polygalacturonase

## Abstract

This paper adopts the Design-Expert software to design an orthogonal experiment with a pulse voltage amplitude of 30 kV, processing time of three minutes, and a pulse width of 45 μs as the center points, in order to study the effects of the pulsed electric field on the cell wall and infection activity of *Rhizoctonia solani*. High-voltage pulse power was used to treat the bacteria solution with the pulsed electric field. Untreated *Rhizoctonia solani* were used as the control group. Transmission electron microscope images were used to analyze the cell wall damage. ANOVA was performed on the experimental results and the fitting degree of the model was good (F>>1). Response surface analysis was used to optimize the parameters based on chitin content and polygalacturonase activity. The optimal treatment conditions were obtained as a pulse voltage amplitude of 25 kV, processing time of 2.54 min, and a pulse width of 34.35 μs. On this basis, experiments were designed to verify the optimized conditions. The results demonstrated that, under the optimal processing conditions, the damage index of the cell wall of *Rhizoctonia solani* was 9.59% lower in chitin content and 83.05% lower in polygalacturonase activity compared with those of the control group. All indexes were significantly different (P < 0.001), which is consistent with the parameter optimization results. The results provide a theoretical basis for the pulsed electric field assisted sterilization and reference for the design of plant protection machinery in the latter stage.

## 1. Introduction

Rice sheath blight is widely disseminated in various rice regions around the world [1,2]. At present, it is listed as the major disease of rice, which is a serious obstacle to a high and stable production yield [3]. After the onset of sheath blight, the rice leaves wither, the seed setting rate and thousand-grain weight decrease, and the increased number of blighted grains result in a yield loss of 10%–30%, which may reach up to 50% in severe cases [4]. It is mainly caused by the infection of *Rhizoctonia solani* (*R. solani*) Kuhn under high-temperature and high-humidity conditions. When the field temperature is more than 25 °C, the relative humidity is more than 90%, and the spread of disease accelerates, having a highly competitive saprophytic ability [5].

There are no rice varieties that are completely immune to sheath blight, while resistant varieties are very rare. Jia Y et al. [6] screened and constructed resistance genes to control rice sheath blight, but the safety of genetically modified food caused by the resistance genes was questioned by consumers. Nowadays, the mainstream control method is chemical control using validamycin, methyl, and other drugs, however, the environmental pollution and fungal resistance caused by long-term use should not be overlooked [7]. Fengyu Shi et al. [8] made some progress in biological control by using the antagonistic action of *Trichodema longibrachia-tun* in order to suppress *R. solani*. However, the difficult screening of antagonistic microorganisms, the constraints of biocontrol fungus by a variety of factors, and the unstable control effect are common problems in the large-scale use of biological control.

The high-voltage pulsed electric field (PEF) has been considered as a new physical bactericidal method by many researchers at home and abroad [9]. An instantaneous high voltage is generated between two electrodes and a PEF acts on the fungus, damaging the physical structure of the fungus and causing the fungal cell contents to overflow, thus leading to the apoptosis or death of the cells. This method has the advantages of being green and pollution-free and is appropriate for the sterilization of crops.

The inactivation effect of PEF on microorganisms depends on the species and the physiological characteristics of the treated objects. Among them, the cell wall of fungi has structures, such as the apical sac, epidermis, spore coat, and the outer cell wall, and the water content of its components is small. The superimposed properties of the fungal cell give them a better insulation capacity [10], which has a great impact on the sterilization effect of PEF. In a study on the inactivation of mold ascospores and conidiospores in apple juice by high-voltage PEF, Raso et al. [11] found that mold conidiospores were very sensitive to PEF. More specifically, under the action of eight pulses at 41.7 kV/cm, the survival decreased by six log cycles. However, the fisheri ascospores did not become inactivated after a PEF treatment of 40 pulses at up to 51 kV/cm. The fisheri ascospores might have a very thick intermediate space in the cell wall that strengthens the resistance to PEF. The precise reasons require further study. As a filamentous fungus, the cell wall thickness of *R. solani* is about 100–250nm, which has a protective effect on cells and is not easy to be destroyed when subjected to high-voltage PEF treatment. Currently, no studies on the damaging effect of PEF on the cell wall of *R. solani* have been reported.

Rice sheath blight disease forms by germinating mycelium under suitable conditions and infection through the stomata or epidermis of the rice leaves. Narrowing the activity of rice sheath blight infection can effectively reduce the formation and spread of the disease. Ming-hai LI et al. [12] processed a *R. solani* fungus solution using a validamycin solution with mass concentrations of 20, 100, and 500 ug/mL. Based on the fact that the lower the PG activity, the lower the ability to infect, they found that, when the concentration was 20 μg/mL, the decrease in polygalacturonase (PG) activity was not obvious, when the concentration increased to 100 μg/mL, the PG activity decreased significantly, and when the concentration was greater than 100 μg/mL, there was no obvious difference in PG activity between the groups. The physical structure of the fungus was damaged by PEF, which should have a certain effect on their infection activity.

In view of the current research status, in this paper, PEF was applied to *R. solani* cells. The damage of the cell wall under high-voltage PEF was observed by projection electron microscopy and was quantitatively analyzed according to the change in chitin content, while the PG activity was detected to verify the effect of PEF on the infection activity of *R. solani*. This study lays a foundation for the research of PEF sterilization.

## 2. Materials and Methods

### 2.1. Materials and Reagents

Strains of *R. solani* were provided by the College of Food Science and Technology, Nanjing Agricultural University. They were cultured in Potato Dextrose Agar medium at 28 °C and were placed in 4 °C refrigerators after 72 h cryopreserved spare. The reagents used in the experiment are listed in Table 1.

### 2.2 Experimental Method

#### 2.2.1. Preparation of Bacterial Solution

First, solid mycelium cryopreserved in a 4 °C refrigerator were removed and were let to recover for two hours in a constant temperature incubator (FYL-YS-151L) of 28 °C. Two bacterial cakes with a diameter of 5 mm were inoculated into a triangular flask containing 150 mL of potato glucose medium sterilized in a High-pressure sterilization pot (BSD-250). Then, they were removed and blew evenly after 72 h of cultivation at 28 °C in an incubator with a rotating speed of 175 rpm. Next, the conductivity was adjusted to 1200 μs/cm with distilled water. The whole process was performed under sterile conditions.

#### 2.2.2. PEF Treatment

**Figure 1:**
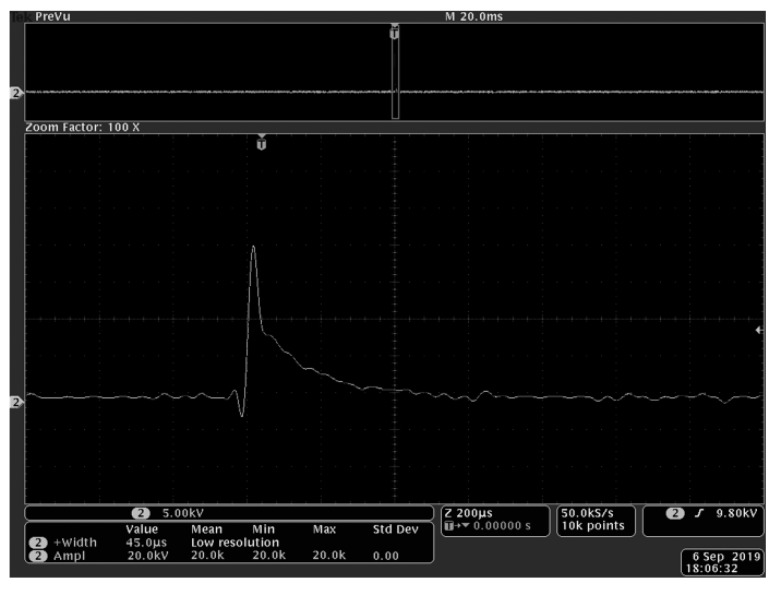
The voltage waveform recorded at center point of 20 kV, 45 μs.

The length, width, and height of the process chamber used to hold the fungus solution of *R. solani* were 50mm, 10mm, and 45mm, respectively. The cells could be damaged during the PEF treatment, leading to a change in conductivity. Therefore, the output amplitude and pulse width of the laboratory custom-made high-voltage PEF generator observed by oscilloscope (Tektronix DPO4104) were adjusted to meet the set experimental conditions by using saline water with 1200 μs/cm conductivity as the substitute. After the adjustment, the samples were processed, and the processes were timed. Each condition was replicated three times. Based on the preliminary exploration of the previous experiment, an output pulse voltage amplitude of 15 ~ 25 kV and a pulse width of 30 ~ 60 μs were selected. A pulse frequency of 2 Hz, processing time of 1 ~ 5 min, with 20 kV, 45 μs and 3 min as the center point. The voltage waveform recorded at the center point is shown in Figure 1 and is attenuated 1000 times by the voltage divider Tektronix P6015A. Orthogonal experiments were designed and analyzed by Design-Expert in order to verify the influence of the above three factors on the fungal cell wall. The factor levels are shown in Table 2. At the same time, the control group was set without PEF treatment.

#### 2.2.3. TEM Examination

The method of “double fixation with glutaraldehyde and osmium acid" was used. 1.5 mL of bacteria solution and 2.5% glutaraldehyde solution were added in a 2 mL centrifuge tube and were fixed for 2 h at 4 °C. After adding 1% osmic acid solution in the fume hood they were fixed 2 h. The fixator was removed by using Centrifuge (Hongke Technology, Jintan, China) and rinsed three times with 0.1 mol/L phosphate buffer with pH = 7.0, 15 min each time. Then, ethanol solution with gradient concentration (50%, 70%, 80%, 90%, and 95%) was used for dehydration. The sample was processed with each concentration for 15 min and was then treated with 100% ethanol for 20 min. After centrifugation, the supernatant was discarded. The sample was placed in a dryer at room temperature overnight. The LKB2088 V type ultra-thin slicing machine was used to cut the sample into slices. A conductive adhesive was used to adhere the gold spraying for 3 min, and the samples were collected and placed under the electron microscope for observation. Dry untreated samples were used as the control.

#### 2.2.4 Determination of Chitin Content

The treated mycelium was collected, and the medium attached to the mycelium was washed and filtered repeatedly with sterile deionized water in the Ultra-clean table (JD-CJ-2S). The mycelia were dried at 90 °C to constant weight, and then the control sample and the processed samples (m_1_) were weighed. The sample was put into the digestive tube containing a saturated KOH solution, which was placed in a constant temperature oil bath pot (GYY, Shanghai Shuangshun Industrial Development Co., Ltd, Shanghai, China) with methyl silicone oil for 1 h at 160 °C. Dehydration was carried out with 95% and 100% alcohol. After dehydration, the samples were weighed by Analytical-balance (BSM-120.4) and m_2_ was obtained [13]:

(1) $$C_{\mathrm{chitin}}\left(\%\right)=\frac{m_{2}}{m_{1}}\times 1.26\times 100\%,$$

where *C_chitin_* (%) is the content of chitin in the cells of *R. solani*, *m*_1_ (g) is the weight of mycelium dried at 90 °C to constant weight, and *m*_2_ (g) is the weight of the sample after dehydration with 95% and 100% alcohol.

#### 2.2.5. Determination of PG Activity

Mycelium treated with high-voltage PEF was repeatedly rinsed with sterile deionized. The culture medium was washed and filtered in order to extract the mycelium, 0.1 g of which were accurately weighed. The mycelia were added into 3 mL of precooled 0.05 mol/L acetic acid-sodium acetate buffer (pH 5.5), homogenated in the ice bath for 1 h, centrifuged at a speed of 4000 rpm for 20 min, and the obtained supernatant was the enzyme solution to be tested. Then, 0.5 mL of 0.05 mol/L acetic acid-sodium acetate buffer solution (pH 5.5), 2.5 mL liquid enzymes under test, and 0.5 mL of 10 g/L of galacturonic acid polymer solution were added to a test tube. After reacting for 30 min at 37 °C, the sample was removed, and 0.3 mL of DNS reagent was immediately added to terminate the reaction. Distilled water was used to dilute the solution to 10 ml. Then, the absorbance was measured by Spectrophotometer (UV752N, Shanghai Youke Instruments Limited, Shanghai, China) at a 540 nm wavelength.

## 3. Results

### 3.1. Effects of PEF Treatment on the Cell Wall of R. solani

**Figure 2:**
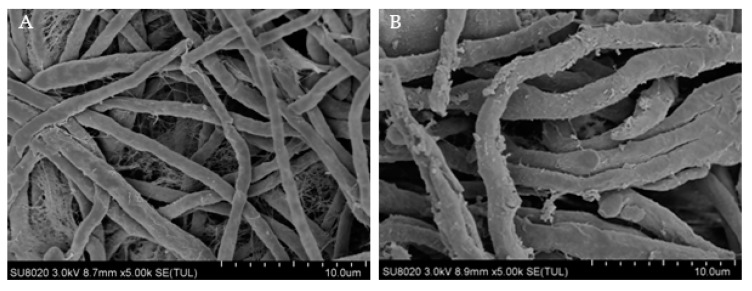
Effects of PEF treatment on the cell wall of *R. solani*. (**A**) TEM images of *R. solani* untreated. with PEF. (**B**) TEM images of *R. solani* treated with PEF.

During the experiment, it was observed that, after centrifugation of the treated bacterial solution, the mycelium volume was smaller than control group. Thus, it demonstrated that the morphology of the *R. solani* cells treated with PEF was damaged. The cell wall, as an important structure to maintain cell morphology, was inevitably damaged. Transmission electron microscope (TEM JEM-1200EX (120 kV), Jeol, Raleigh, NC, United States) morphologies of the treated *R. solani* were shown in Figure 2A,B.

Figure 2A shows the TEM image of *R. solani* without PEF treatment. It can be observed that the appearance of mycelia was regular and complete, the distribution was even, and the surface of mycelia was smooth and flat without cracks. Figure 2B shows the TEM image of *R. solani* after PEF treatment. The visible mycelium phenotype was significantly changed, and the surface of the mycelium was uneven, with uneven cracks and a large number of buds. According to this, the cell membrane may be partially damaged, which may lead to the overflow of cell contents, and if the overflow exceeds a certain threshold, it may lead to apoptosis or death.

### 3.2. Effect of PEF Treatment on the Infection Activity of R. solani Cells

In the orthogonal test, there were 17 treatments with five replications at the central value point. The experimental results are given in Table 3.

The original data listed in Table 3 were obtained after the results in the three experimental groups were averaged and significance analysis was carried out.

It can be seen from Table 3 that most of the chitin content and PG activity after PEF treatment were significantly lower than CK. Only when the treatment parameters were 20 kV, 30 μs, 1 min, and 15 kV, 30 μs, 3 min, were the effects of PEF treatment on *R. solani* insignificant.

Design-Expert was used to perform multiple regression fitting on the test data in Table 4 and a quadratic multiple regression model was established among chitin content, PG activity, pulse voltage amplitude, pulse width, and processing time. Variance analysis was performed on each index and the analytical results are shown in Table 4.

### 3.3. Effect of High-Voltage PEF Treatment on Chitin Content in R. solani

It can be seen from Table 4 that after the PEF treatment, the content of chitin in *R. solani* cells was significantly lower than the control group (P < 0.05) and an extremely significant difference (P < 0.001). However, there were not any significant changes in either group. A quadratic multiple regression model between the content of chitin and electric field parameters was established through response surface analysis, as showed in Equation (2):

(2) $$C_{\mathrm{chitin}}=0.26518-0.0027\cdot PW-0.011863\cdot t-0.016238\cdot V-0.00115\cdot t\cdot PW+0.0022\cdot V\cdot PW\;\;-0.007725\cdot V\cdot t+0.0047725\cdot {PW}^{2}+0.0041975\cdot t^{2}+0.0003475\cdot V^{2},$$

where *C_chitin_* (%) is the content of chitin in the cells of *R. solani* treated with PEF and was used to measure the effect of different treatment conditions on the cell wall infection activity of *R. solani*; *V* (kV) is the pulse voltage amplitude; *T* (min) is the processing time; and PW (μs) is the pulse width.

According to the data presented in Table 4, the model had an F value of 87.57 and an R^2^ of 0.9912. The fitting degree of the model was good, and the accuracy and reliability of the experiment were high.

Based on the analysis of the data in Table 4, the effects of pulse voltage amplitude, pulse width, and processing time on chitin content were all significant (P < 0.01). The correlation between pulse voltage amplitude and processing time demonstrated a significant impact (P < 0.01), while no significant correlation was found between pulse voltage amplitude and pulse width, and between pulse width and processing time on chitin content (P > 0.05). 

#### 3.3.1. Response Surface Analysis of the Correlation between Pulse Voltage Amplitude and Processing Time on chitin Content

**Figure 3:**
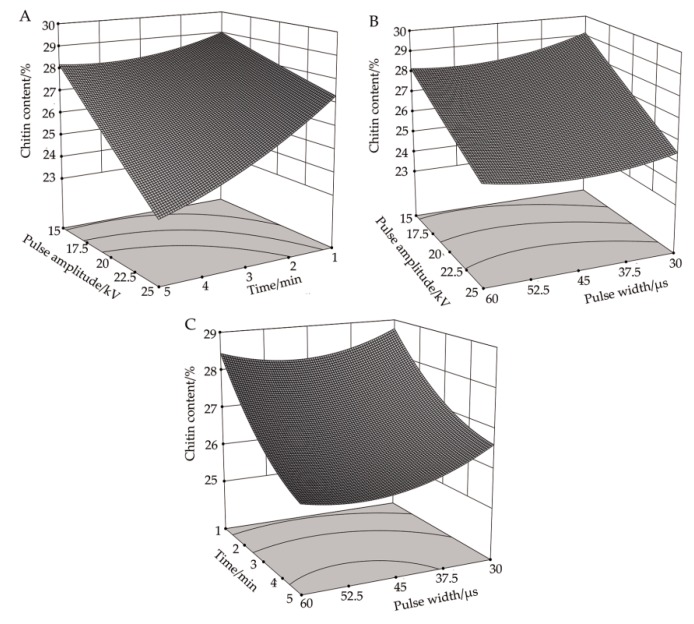
Effects of high-voltage PEF treatment on chitin content in *R. solani*. (**A**) Time-amplitude response surface analysis. (**B**) Pulse width-amplitude response surface analysis. (**C**) Pulse width-time response surface analysis.

Figure 3A illustrates the response surface plot of pulse voltage amplitude and processing time to the content of chitin in *R. solani* cells when the pulse width was 45 μs. It can be seen that, when the pulse width was at its central value, the effects of increasing the pulse voltage amplitude and the processing time on the chitin content were similar. In particular, as these factors were increasing, the content of chitin decreased, and the changing trend was negatively correlated with the processing conditions. It can be seen from Table 4 that the correlation between pulse voltage amplitude and processing time on chitin content was significant (P < 0.01), which was confirmed by the response analysis in Figure 3A. When the pulse voltage amplitude was 25 kV and the treatment time was five minutes, the chitin content reached a minimum value of 23.39%, which was 19.34% lower than in the control group.

#### 3.3.2. Response Surface Analysis of the Correlation between Pulse Voltage Amplitude and Pulse Width on Chitin Content

Figure 3B shows the response surface plot of pulse width and pulse voltage amplitude to chitin content in *R. solani* cells when the processing time was three minutes. It can be seen that, when the processing time was at its central value, the effect of increasing the pulse voltage amplitude on chitin content was the same under a different pulse width, and both factors demonstrated a decreasing trend. Under a different pulse voltage amplitude, the effect of increasing the pulse width on chitin content was also similar, which at first decreased slowly and then increased slightly. When the pulse voltage amplitude was 25 kV and the pulse width was 45.8 μs, the content of chitin reached a minimum value of 24.93%, which was 14.03% lower than in the control group.

#### 3.3.3. Response Surface Analysis of the Correlation between Pulse Width and Processing Time on Chitin Content

Figure 3C shows the response surface plot of pulse width and processing time to the chitin content in *R. solani* cells when the pulse voltage amplitude was 20 kV. It can be seen that when the pulse voltage amplitude was at its central value, the chitin content decreased with different pulse widths as the processing time increased. With the increase of pulse width, the content of chitin at first decreased gradually and then leveled off. The effects of pulse width and processing time on chitin content were similar and demonstrated a negative correlation. When the pulse width was 51.9 μs and the treatment time was five minutes, the chitin content reached a minimum value of 25.68%, which was 11.45% lower than in the control group.

### 3.4. Effect of High-Voltage PEF Treatment on PG Activity 

It can be seen from Table 3 that, after PEF treatment, the activity of most dry mycelium samples of *R. solani* against PG of CK had significant (P < 0.05) or (P < 0.01) differences, with no significant changes in the individual groups. A quadratic multiple regression model between PG activity and electric field parameters was established through response surface analysis, and is shown in Equation (3):

(3) $$A_{\mathrm{PG}}=0.0158-0.00525\cdot PW-0.00925\cdot t-0.0205\cdot V+0.0055\cdot t\cdot PW+0.001\cdot V\cdot PW\;\;+0.005\cdot V\cdot t+0.00935\cdot {PW}^{2}+0.00535\cdot t^{2}+0.00385\cdot V^{2},$$

where *A_PG_* is the PG activity in *R. solani* cells that measures the influence of different treatment conditions on the cell wall infection activity of *R. solani*, and its unit is the enzyme activity unit (U) [14]. The largest effect on this index had a pulse voltage amplitude, and the least impact had the cross term between pulse voltage amplitude and pulse width.

It can be seen from Table 4 that the model’s F value was 41.38 and R^2^ was 0.9815, indicating that the established expression of the relationship between PG activity and the various experimental factors was extremely significant (P < 0.01). The model had a good fitting degree and the accuracy and reliability of the experiment were high.

Based on the data presented in Table 4, the effects of pulse voltage amplitude, pulse width, and processing time on chitin content were all found to be significant (P < 0.01). The correlation between pulse voltage amplitude and processing time was proven to significantly affect PG activity (P < 0.05). The correlation between pulse voltage amplitude and pulse width had no significant effect on PG activity (P > 0.05).

#### 3.4.1. Response Surface Analysis of the Correlation between Pulse Voltage Amplitude and Processing Time on PG Activity

**Figure 4:**
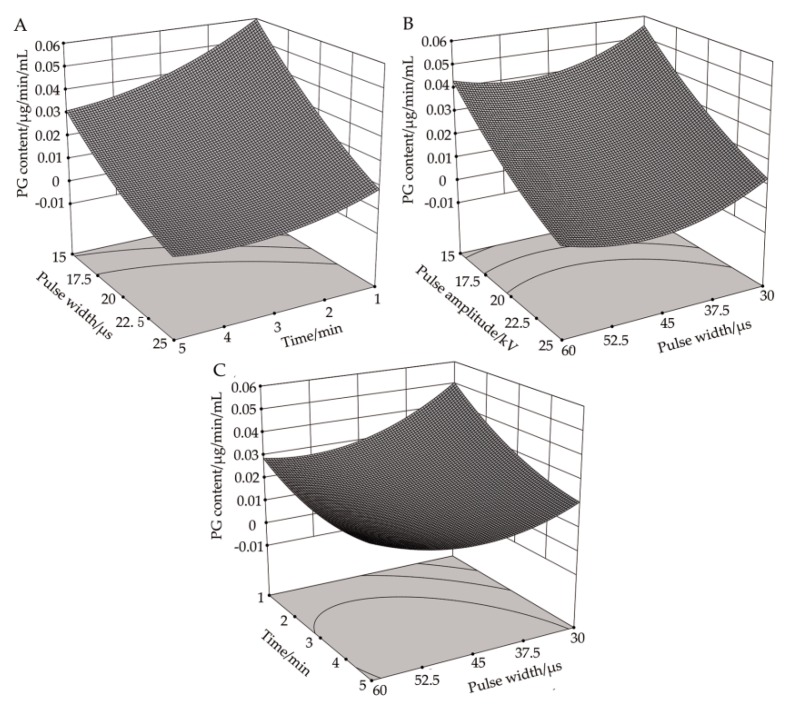
Effects of high-voltage PEF treatment on PG activity in *R. solani*. (**A**) Time-amplitude response surface analysis. (**B**) Pulse width-amplitude response surface analysis. (**C**) Pulse width-time response surface analysis.

Figure 4A shows the response surface plot of pulse voltage amplitude and processing time to PG activity in *R. solani* cells at a pulse width of 45 μs. It can be seen that, when the pulse width was at its center value, the impact of increasing the pulse voltage amplitude on PG activity was similar to that of increasing the pulse voltage amplitude at different processing times. The PG activity decreased, and the changing trend was negatively correlated with it. At different pulse voltage amplitudes, the PG activity at first decreased slowly and then increased slightly with the increasing treatment time. It can be seen from Table 4 that the correlation between pulse voltage amplitude and processing time on PG activity was significant (P < 0.05), which was also confirmed by the response analysis plot in Figure 4. When the pulse voltage amplitude was 25 kV and the processing time was 3.89 min, the theoretical value of PG activity is less than 0, suggesting that PG was completely deactivated and that the correlation between pulse voltage amplitude and processing time has a significant impact on PG activity.

#### 3.4.2. Response Surface Analysis of the Correlation between Pulse Voltage Amplitude and Pulse Width on PG Activity

Figure 4B demonstrates the response surface plot of pulse voltage amplitude and pulse width to PG activity in *R. solani* cells when the processing time was three minutes. It can be seen that, when the processing time was at its central value, the influence of increasing the pulse voltage amplitude on PG activity was the same under different pulse widths, and both factors demonstrated a decreasing trend. At different pulse voltage amplitudes, the effect of increasing the pulse width on PG activity was also similar, which at first decreased slowly and then increased slightly. When the pulse voltage amplitude was 25 kV and the pulse width was 48.405 μs, the theoretical value of PG activity was less than 0, indicating that PG was completely inactivated.

#### 3.4.3. Response Surface Analysis of the Correlation between Pulse Width and Processing Time on PG Activity

Figure 4C shows the response surface plot of pulse width and processing time to PG activity when the pulse voltage amplitude was 20 kV. It can be seen that, when the pulse voltage amplitude was at its central value, the PG activity decreased with the increase of processing time under different pulse widths, and the two factors exhibited a negative correlation trend. With the increase of pulse width, the PG activity at first decreased gradually and then increased slowly at different treatment time points. When the pulse width was 45.47 μs and the processing time was 4.70 min, the PG activity reached a minimum value of 0.01179, which was 80.02% lower than that in the control group.

### 3.5. Parameter Optimization

The minimum values of the chitin content and PG activity of *R. solani* cells after PEF treatment were used as the optimization objective. The weight of the two indexes was 1. A response surface analysis method was used to optimize the parameters of the fitted mathematical model and the optimal combination of treatment conditions for *R. solani* under high-voltage PEF was obtained: pulse voltage amplitude of 25 kV, pulse width of 34.35 μs, and processing time of 2.54 min. Under the optimal conditions, the expected values of chitin content and PG activity in *R. solani* were 25.66% and 0.00904311, respectively, which were significantly lower than those in the control group.

### 3.6. Experimental Verification

Based on the parameter optimization results, the pulse voltage amplitude of 25 kV, pulse width of 34.35 μs, and processing time of 2.54 min were applied as treatment conditions in a high-voltage PEF treatment on the bacterial solution of *R. solani*. The chitin content and PG activity of *R. solani* were found to be 26.22% and 0.01, respectively. Compared to the control group, the chitin content and PG activity in cell wall damage indexes decreased by 9.59% and 83.05%, respectively. All indexes demonstrated a significant difference (P < 0.001), which was in agreement with the parameter optimization results.

## 4. Discussion

The electric field is ubiquitous in nature. Organisms on the Earth’s surface live and reproduce under the action of the natural electrostatic field with an electric field intensity of 130 V/m. The internal electric field and the natural electrostatic field form a relatively stable electrostatic system. However, an applied pulsed electric field may disturb the electrostatic balance in cells and cause a series of electrobiological effects [15]. Under high-voltage PEF, the TEM image of *R. solani* cells showed that the mycelium cell wall was significantly damaged, the content of chitin in the cell wall was significantly decreased, and the inactivation degree of PG was significantly affected by the PEF process parameters. When the pulse voltage was 25 KV, the pulse width was 34.35 μs, and the processing time was 2.54 min, all the indexes of *R. solani* reached their minimum value.

The cell wall of fungi is about 100~250 nm thick, accounting for 30% of the dry matter of cells. Its main component is chitin, which is structurally stable and insoluble in water, alcohol, weak acid, weak base, and other liquids. As a fibrous substance [16], it can improve the mechanical strength of the cell wall and has a protective function for cells [17]. Roberts et al. [18] measured the content of chitin and fungi in the tissue of tall fescue, and obtained a calibration coefficient and a variation coefficient of 0.86 and 0.84, respectively, which indicated a high correlation between the content of chitin and the infection activity of endophytic fungi. In the present study, the chitin content was selected as the standard for cell wall destruction.

The response surface analysis results showed that the content of chitin decreased significantly with the increase of pulse voltage amplitude and processing time (P < 0.01). More specifically, at first, it decreased sharply and then recovered slowly with the increase of pulse width—however the effect was not significant. This is similar to the result obtained through the analysis of theoretical formula (4) [19]:

(4) $$W_{0}=\frac{U_{0}^{2}}{R}\cdot \tau \cdot ƒ\cdot t\;\;,\;\;$$

where *W_0_* is the pulse energy; U_0_ is the electrode voltage; *R* is the total resistance of the load circuit; *τ* is the pulse width; *ƒ* is pulse frequency; and *t* is the processing time. Clearly, *W_0_* is directly proportional to the quadratic term of the pulse voltage amplitude, directly proportional to the pulse width and processing time, and inversely proportional to the load resistance.

In the correlation between pulse voltage amplitude and processing time, the pulse energy at the extreme value increased by 13.89 times. In the correlation between pulse width and processing time, the pulse energy at the extreme value was increased by 10 times. In the correlation between pulse voltage amplitude and pulse width, the pulse energy at the extreme value was increased by 5.56 times. In terms of the damaging effect of PEF on the cell wall of *R. solani*, the correlation effect between pulse voltage amplitude and processing time was significant, which was consistent with the conclusion of this experiment.

At present, there is a lack of research on the destructive effect of PEF on the cell wall of *R. solani*. The present study aimed at addressing current deficiencies, and thus, the content of chitin in the cell wall under different PEF process parameters was experimentally analyzed. When the pulse voltage was 25 kV and the processing time was five minutes, the content of chitin reached a minimum value of 23.39%, which was 19.34% lower than that of the control group, indicating that the cell wall damage was serious and the sterilization effect was remarkable. Ann E. Russell’s research has demonstrated that the major constituent of cell walls of soil fungi, chitin, can decompose rapidly and release substantial N that is available for plant and microbial growth [20], and this result may be helpful to improve the yield of grain. However, the specific decomposition products of chitin after PEF treatment need to be further verified by experiments.

Pectinase plays a major role in pathogen invasion and host adaptation [21]. The main diseases caused by pectinase are soft rot, leaf spot, and vascular bundle disease. In soft rot tissue, if phenols are oxidized to quinones, they become brown rot [22]. For example, when Rhizopus stolonifer infected melon, mycelia secreted large amounts of PME (Pectin methylesterase), PG, and PMG (Polymethylgalacturonase), which could rapidly dissolve the gelatinous layer of the tissue and induce electrolyte extravasation, plasmolysis, and soft rot. The PG activity in fungal cells has been linked to the decomposition of cell walls. When the PG activity in cells increases, the cells are in senescence, and the cell walls begin to decompose [23]. Studies have demonstrated that cell wall degrading enzymes are main pathogenic factors and have a significant pathogenic effect [24]. Ming-hai LI et al. [25] confirmed that there is a positive correlation between the infection activity of *R. solani* and the activity of cell wall degrading enzymes. PG is a type of cell wall degrading enzyme, which is highly active in normal cells and its activity is significantly reduced after PEF treatment. Therefore, in this experiment, the PG activity changes in cells were utilized to reflect the infection activity of *R. solani*.

Based on the experimental results, the processing time and pulse voltage amplitude exhibited significant effects on PG. In a study of PG inactivation by PEF, Chen Yi et al. [26] found that when the amplitude of pulse voltage was 20 kV/cm and the number of pulses increased from 30 gradients to 270 (increase in processing time), the PG activity decreased from 78.9% to 31.2%, and the passivation effect of PG was significantly enhanced, reflecting that the cell infection activity was significantly affected (P < 0.01). In the present study, when the PEF process parameters were pulse voltage amplitude of 30 kV, processing time of three minutes, and pulse width of 45 μs the PG activity reached a minimum value of 1.179%. Compared to the results of Chen Yi et al. [1], the PG activity was significantly reduced, reflecting a significant attenuation in the infection activity of *R. solani*, while indicating that the PEF process parameters in this experiment were better. When the pulse voltage amplitude was 25 kV, the PG was deactivated after 3.89 min. An increase in pulse voltage amplitude shortened the critical deactivation time, which is in agreement with the experimental conclusion of Andreou et al. [27]. In the same study it was found that, under PEF process parameters of 12.5 kV/cm and 4.5 ms, the PG activity was 20%, and the experimental results varied considerably. The passivation effect was significantly worse than that of this study. Aguilo-Aguayo et al. [28] inactivated PG with PEF of 35 kV/cm for one minute, and the activity changed to 14% after storage for one day. On the contrary, in our study, the PG activity was significantly reduced by increasing the processing time and was completely inactivated at 25 kV/cm, which improved the energy utilization rate and effectively reduced the cell infection activity.

In addition, the factors influencing PG activity are diverse. Zhaowei et al. [29] investigated the passivation effect of PEF on different enzymes and found that higher temperatures also speed up the inactivation of enzymes. Under process parameters of 30 kV/cm and 1200 μs, and a temperature increase from 10 °C to 40 °C, the residual enzyme activity was reduced by 20.1%. When the electric intensity was changed, the enzyme activity fluctuated slightly. It was concluded that temperature plays a dominant role among the factors affecting enzyme activity. Tian Hongyun et al. [30] suggested that a certain intensity of PEF affected the three-dimensional structure of the enzyme protein and inhibited the activity of the enzyme. When the electric field reached a certain intensity, the structure changed dramatically, which denatured the enzyme and significantly reduced its activity. At present, relevant theoretical explanations are not complete, and the specific reasons need to be further investigated. The effect of pulse width on *R. solani* was found not significant in this experiment, which was supposed to be related to the small increment of pulse width condition.

Furthermore, it should be mentioned that the emergence of the PEF sterilization process has decreased the use of drugs in production and living, which has improved food safety. In addition, further research on PEF sterilization is required, in order to provide a theoretical basis for the construction of agricultural facilities and the design of rice plant protection machinery. With the increasing demand for a higher quality of life, this process will inevitably have broader market application prospects.

## 5. Conclusions

(1) TEM image analysis demonstrated that, after PEF treatment, the mycelia phenotype of *R. solani* changes significantly, and the cell contents escape and adhere to the surface layer. This indicates that PEF with a certain strength could effectively destroy the cell wall.

(2) According to the experimental data, we speculated that the correlation between pulse width and treatment time may have a significant effect on PG activity (P < 0.01). However, when the pulse voltage amplitude was changed, the PG activity was theoretically calculated to be less than 0 and the deactivation degree was relatively large. The specific factors need to be experimentally verified.

(3) The experimental determination of chitin content was used to measure the degree of cell wall damage. Response surface analysis results demonstrated that the correlation between pulse voltage amplitude and processing time on chitin content was significant (P < 0.01), which was consistent with the experimental data. However, the effects of pulse width and processing time were not found to be significant (P > 0.05).

(4) A response surface analysis method was used to optimize the processing conditions, and the optimal electric field parameters were found: pulse voltage amplitude of 25 kV, pulse width of 34.35 μs, and processing time of 2.54 min. Under these process parameters, the experimental values of chitin content and PG activity in the bacterial solution were 26.22% and 0.010, respectively. Compared with CK, the chitin content was reduced by 9.59% and the PG activity was reduced by 83.05%, which were significantly lower than those of CK (P < 0.01). 

## Figures and Tables

**Table 1:** Reagents used for experiment.

Reagent Name	Reagent Parameter
KOH	100%
Polygalacturonic acid	10 g/L
Acetic acid-sodium acetate buffer	0.05 mol/L, pH=5.5
DNS	—
Glutaraldehyde solution	2.5%
Osmic acid solution	1%
Phosphate buffer	0.1 m, pH = 7.0
Ethanol	50%, 70%, 80%, 90%, 95%, 100%
Acetone	100%

**Table 2:** Factors and levels of the orthogonal PEF experiments.

Level	Factors		
	Pulse Width (μs)	Processing Time (min)	Pulse Voltage Amplitude (kV)
-1	30	1	15
0	45	3	20
1	60	5	25

**Table 3:** Result of orthogonal experiment of PEF.

Test NO.	Pulse Width (μs)	Processing Time (min)	Pulse Voltage Amplitude (kV)	PG Activity (U)	Chitin Content (%)
Control check	0	0	0	0.059	29
1	30	5	20	0.024**	26.53**
2	45	3	20	0.016**	26.59**
3	45	1	25	0.012**	27.12**
4	45	5	25	0.002**	23.26**
5	45	3	20	0.016**	26.41**
6	60	5	20	0.02**	25.87**
7	30	3	25	0.008**	25.67**
8	45	3	20	0.016**	26.75**
9	30	1	20	0.052^ns^	28.73^ns^
10	45	3	20	0.015**	26.35**
11	45	1	15	0.058**	29.14^ns^
12	60	3	15	0.048**	27.95^ns^
13	45	3	20	0.016**	26.49**
14	45	5	15	0.028**	28.37**
15	60	3	25	0.004**	25.46**
16	60	1	20	0.026**	28.53^ns^
17	30	3	15	0.056^ns^	29.04^ns^

Note: ‘*’ in the table represents significance as P < 0.05, ‘**’ in the table represents extremely significance as P < 0.01, ‘ns’ in the table represents non-significance as P > 0.05.

**Table 4:** Response surface ANOVA results.

Source of Variation	Chitin Content		PG Activity	
	F value	P value	F value	P value
Model	87.57	<0.0001	41.38	<0.0001
V	444.58	<0.0001	245.66	<0.0001
PW	12.29	0.0099	16.11	0.0051
t	237.28	<0.0001	50.02	0.0002
V·t	50.31	0.0002	7.31	0.0305
V·PW	4.08	0.0831	0.29	0.6055
PW·t	1.12	0.3216	8.84	0.0207
V^2^	0.11	0.7530	4.56	0.0701
t^2^	15.64	0.0055	8.81	0.0209
PW^2^	20.21	0.0028	26.90	0.0013
R^2^	0.9912		0.9815	

Note: V, pulse voltage amplitude; PW, pulse width; t, processing time; R, R-Squared.

## References

[1] Rabindran R., Vidhyasekaran P. (1996). Development of a formulation of Pseudomonas fluorescens PfALR2 for management of rice sheath blight. Crop Prot..

[2] Zuo S., Zhang Y., Yin Y., Li G., Zhang G., Wang H., Chen Z., Pan X. (2014). Fine-mapping of qSB-9 TQ, a gene conferring major quantitative resistance to rice sheath blight. Mol. Breed..

[3] Liu W., Yang C. (2009). Research progress on biological control of rice sheath blight. Guangxi Agric. Sci..

[4] Meng Q.Z., Liu Z.H. (2001). Research progress of rice sheath blight. Shen Yang Agric. Univ..

[5] Lore J.S., Hunjan M.S., Singh P., Willocquet L., Sri S., Savary S. (2013). Phenotyping of Partial Physiological Resistance to Rice Sheath Blight. J. Phytopathol..

[6] Eizenga G.C., Agrama H.A., Lee F.N., Yan W., Jia Y. (2006). Identifying Novel Resistance Genes in Newly Introduced Blast Resistant Rice Germplasm. Crop Sci..

[7] Hu X.R. (2006). The Resistance Monitoring of Rhizoctonia Solani Tojinggangmycin and Its Resistance Risk Assessment.

[8] Shi F.Y., Zhu Y.D. (2005). Antagonistic effect of trichoderma elongata T8 on rice sheath blight. Chin. Agric. Sci. Bull..

[9] Novickij V., Lastauskiene E., Staigvila G., Girkontaite I., Zinkeviciene A., Svediene J., Paskevicius A., Markovskaja S., Novickij J. (2019). Low concentrations of acetic and formic acids enhance the inactivation of Staphylococcus aureus and Pseudomonas aeruginosa with pulsed electric fields. BMC Microbiol..

[10] Fang T., Yan Z.M. (2007). Studies on lethal dynamics of S. accha rom ycesce revisiae E.coliand penic illium by treatment time of pulse electric field. Food Mechinery.

[11] Raso J., Calderón M.L., Gongora M., Barbosa-Canovas G., Swanson B.G. (1998). Inactivation of Mold Ascospores and Conidiospores Suspended in Fruit Juices by Pulsed Electric Fields. LWT Food Sci. Technol..

[12] Yang Y.Q., Li M.H., Li Y. (2011). Establishment of Agrobacterium tumefaciens-Mediated Transformation System for Rice Sheath Blight Pathogen *Rhizoctonia solani* AG-1 IA. Rice Sci..

[13] Gong P., Wang J., Liu B., Ru G., Feng J. (2016). Dissolution of chitin in aqueous KOH. Cellulose.

[14] Redgwell R.J., Melton L.D., Brasch D.J. (1992). Cell Wall Dissolution in Ripening Kiwifruit. Plant Physiol..

[15] Xie J.F., Yi W. (2002). The Effect of High Electrostatic Field on Plant Cellular Transmembrane Voltage and Micro Principles. J. Wuhan Inst. Sci. Technol..

[16] Dai Y.Y., Cao J. (2004). Research progress of chitin and chitosan in fungi. J. Zhengzhou Inst. Technol..

[17] O’Brien J.A., Daudi A., Butt V.S., Bolwell G.P. (2012). Reactive oxygen species and their role in plant defence and cell wall metabolism. Planta.

[18] Roberts C.A., Barton F.E., Moore K. (1988). Moore Estimation of Acremonium Coenophialum Mycelium in Infected Tall Fescue. Agron. J..

[19] Li H.T., Deng W.J. (2015). Analysisofinactivation saccharom ycescerevisiae by 13 μs,200 ns and 2 ns pulsed electric fields. Highpower Laser Partilice Beams.

[20] Russell A.E. (2014). Unexpected Effects of Chitin, Cellulose, and Lignin Addition on Soil Dynamics in a Wet Tropical Forest. Ecosystems.

[21] Mao L.C., Zhang S.L. (2001). Role of Pectolytic Enzymes and Cellulase during Ripening and Woolly Breakdown in Peaches. Acta Hortic. Sin..

[22] Bolin H.R., Stafford A.E. (1977). Factors Affecting The Storages Stabilitys of Shredded Lettuce. J. Food Sci..

[23] Qi L.D., Wei J.M. (2015). Pectin POIySaCCharjde Degradation in Relation to the texture Softening in Pear Fruit. Sci. Agric. Sin..

[24] Chen X.J., Zhang H. (2006). Cell Wall Degrading Enzymes Produced by *Rhizoctonia solani* and Their Pathogenicity to Rice Plants. J. Jiangsu Agric..

[25] Khan A., Williams K.L., Nevalainen H.K.M. (2004). Effects of Paecilomyces lilacinus protease and chitinase on the eggshell structures and hatching of Meloidogyne javanica juveniles. Biol. Control.

[26] Chen Y. (2007). Inactivation Mechanics of POD and PG Enzymes in High Voltage Pulsed Electric Field. Master’s Thesis.

[27] Andreou V., Dimopoulos G., Katsaros G., Taoukis P. (2016). Comparison of the application of high pressure and pulsed electric fields technologies on the selective inactivation of endogenous enzymes in tomato products. Innov. Food Sci. Emerg. Technol..

[28] Aguiló-Aguayo I., Soliva-Fortuny R., Martín-Belloso O. (2007). Comparative study on color, viscosity and related enzymes of tomato juice treated by high-intensity pulsed electric fields or heat. Eur. Food Res. Technol..

[29] Zhao J., Zhao W. (2008). Effect of pulsed electric fields on the sterilization and enzyme inactivation of pear juice. Sci. Technol. Food Ind..

[30] Tian H.Y. (2005). Study on the Effect of Pulsed Electric Fields on the Sterilization and Enzyme Inactivation. Master’s Thesis.

